# Lack of full sequencing *GBA1* studies for patients with Parkinson’s disease in Latin America

**DOI:** 10.1038/s41531-022-00358-z

**Published:** 2022-08-08

**Authors:** Bruno Lopes Santos-Lobato, Artur F. Schumacher-Schuh, Ignacio F. Mata

**Affiliations:** 1 Laboratory of Experimental Neuropathology, Federal University of Pará, Belém, PA Brazil; 2 Hospital de Clínicas de Porto Alegre, Porto Alegre, RS Brazil; 3 Genomic Medicine Institute, Lerner Research Institute, Cleveland Clinic, Cleveland, OH USA

**Keywords:** Parkinson's disease, DNA sequencing

## Abstract

Full sequencing of the *GBA1* gene in patients with Parkinson’s disease provides a wide screening of pathogenic variants, but less developed regions of the world, like Latin America, may have difficulties in performing full sequencing. We performed a systematic review with meta-analysis to explore the prevalence and the odds ratio of specific *GBA1* variants in Parkinson’s disease in Latin America. We noted a lack of full sequencing *GBA1* studies in Latin America.

Variants in the *GBA1* gene are strong genetic risk factors for developing sporadic Parkinson’s disease (PD). Some of these variants (p.N370S, p.L444P, and p.E326K) have high allelic frequencies in patients with PD and increased risk for developing the disease^1,2^. The prevalence of *GBA1* variants in Ashkenazi Jewish populations with PD is the highest (18%) compared to other ethnic backgrounds^1^. In non-Ashkenazi patients with PD, the prevalence of *GBA1* variants is close to 10%, but it may vary according to the country and screening methodology.

For *GBA1* analysis, most studies use genotyping methods targeting specific common pathogenic variants or sequencing^3^. *GBA1* is a large gene located in a complex genomic region, with a pseudogene with 96% of homology in the coding sequence, which increases the risk of recombination and poses challenges to PCR and sequencing. Regarding *GBA1* sequencing, while some studies opted for sequencing and analyzing all exons (full sequencing), other groups are sequencing only specific exons, like exons 9 and 10, where common variants are found (targeted sequencing)^3^. Recently, long-read sequencing methods have been employed to study better the region^4^. The full sequencing of *GBA1* warrants the identification of rare or population-specific variants, shedding light on the pathophysiology of effects of *GBA1* in PD and helping to recruit more eligible patients for *GBA1*-targeted trials^5^.

In Latin America, a region with a population of approximately 660 million, few studies on PD associated with *GBA1* variants (PD-*GBA*) have been published. Latinos, referring to those individuals from Latin America, are genetically very heterogeneous due to a complex three-way admixture (Native American, European and African), with large differences in the amounts of each ancestry not only between but also within countries. Furthermore, unfavorable socioeconomic conditions among its countries may hamper the capacity to perform the full sequencing of *GBA1*, which may negatively influence the results of these studies. To explore the prevalence and odds ratio of PD-*GBA* in Latin America, we conducted a systematic literature review and meta-analysis.

A total of 11 clinic-based studies (one study was conducted in two different countries, comprising 12 cohorts) were included in the analysis (Supplementary Fig. 1). A total of 1,719 patients with PD and 1,444 controls were analyzed, and an average prevalence of 5.4% of *GBA1* carriers was found (Table 1). No participant reported Ashkenazi Jewish ancestry. There are some methodological differences among studies: four studies recruited only early-onset patients with PD (age at onset varying from less than 45 to less than 55 years), and two studies did not include healthy controls. Only four cohorts performed full sequencing of *GBA1* (n patients = 735; n controls = 445). Despite Brazil being the most populous country in Latin America and with more patients with PD screened for *GBA1* variants, surprisingly no study from this country used full sequencing of the gene.

**Table 1:** Main characteristics of studies and frequencies of *GBA1* pathogenic variants (plus p.E326K) in patients with PD and controls included in the meta-analysis.

Country and author	Year	Group	*n*	Male sex (%)	AAE	AAO	FH (%)	EOPD (%)	Mutation screened	GBA (%)	L444P (%)	N370S (%)	E326K (%)
Brazil													
Spitz et al.^8^	2008	Patient	65	63.1	54	41	NA	100	N370S, L444P, G777S, E326K	3.07	3.07	0.00	1.54
		Control	267	NA	NA	–				0.00	0.00	0.00	0.00
Socal et al.^9^	2009	Patient	62	59.7	50	41	NA	NA	L444P, N370S, IV2 + 1, 84GG	3.22	1.61	1.61	NA
		Control	NA	NA	NA	–				NA	NA	NA	NA
Santos et al.^10^	2010	Patient	110	67.3	52	41	18.1	100	N370S, L444P, 84GG, IVS2 + 1, G377S	5.45	1.82	1.82	NA
		Control	155	53.0	62	–				0.00	0.00	0.00	NA
Guimarães et al.^11^	2012	Patient	237	62.9	64	57	34.1	23.2	N370S, L444P	3.79	2.11	1.27	NA
		Control	186	51.0	60	–				0.00	0.00	0.00	NA
Abreu et al.^12^	2016	Patient	141	68.1	60	53	100	NA	L444P, N370S	2.83	2.13	0.71	NA
		Control	NA	NA	NA	–				NA	NA	NA	NA
Amaral et al.^13^	2019	Patient	81	61.7	69	55	9.8	NA	L444P, N370S	7.40	3.70	3.70	NA
		Control	81	NA	67	–				0.00	0.00	0.00	NA
Colombia													
Velez-Pardo et al.^7^	2019	Patient	131	48.1	65	49.3	NA	46.5	Full sequencing	11.45	2.29	2.29	1.53
		Control	164	50.0	65	–				2.43	0.00	0.00	0.60
Tipton et al.^16^	2020	Patient	142	52.8	63	53.2	NA	65.4	K198E	2.11	NA	NA	NA
		Control	57	36.0	63	–				1.72	NA	NA	NA
Costa Rica													
Torrealba-Acosta et al.^15^	2021	Patient	118	57.6	62	54.6	17.7	NA	Full sequencing^a^	0.00	0.00	0.00	0.00
		Control	97	28.8	62	–				2.06	1.03	1.03	0.00
México													
González-DelRincón et al.^14^	2013	Patient	128	NA	NA	37.7	NA	NA	L444P, N370S	5.46	5.46	0.00	NA
		Control	252	NA	NA	–				0.00	0.00	0.00	NA
Peru													
Velez-Pardo et al.^7^	2019	Patient	471	54.8	62	57.1	NA	24.2	Full sequencing	5.30	2.76	0.21	1.06
		Control	155	31.8	62	–				1.29	0.64	0.64	0.00
Venezuela													
Eblan et al.^17^	2006	Patient	33	NA	NA	36	NA	100	Full sequencing	12.12	3.03	3.03	0.00
		Control	29	NA	NA	–				0.00	0.00	0.00	0.00

^a^Molecular inversion probes were used for sequencing.

*AAE* age at evaluation, *AAO* age at disease onset, EOPD (%), proportion of patients with early-onset Parkinson’s disease (definition of early-onset Parkinson’s disease varied among studies, from AAO < 45 years to AAO < 55 years); *FH* (%), proportion of patients with positive family history of Parkinson’s disease; GBA (%), total proportion of pathogenic variants of *GBA1* plus p.E326K; *NA* Not available.

We performed a meta-analysis to estimate the odds ratio (OR) of developing PD for carriers of all pathogenic *GBA1* variants plus p.E326K, and specifically for carriers of p.L444P and p.N370 variants. We used a fixed-effect model with a continuity correction of 0.5 for studies with zero *GBA1* carriers in the PD or control group. For calculating the OR, we used the Cochran–Mantel–Haenszel test and the Tarone test to examine heterogeneity^6^. Statistical tests were performed using the R software version 4.0.4, with the package *metafor*.

In the meta-analysis to estimate the OR of all pathogenic *GBA1* variants (p.L444P, p.N370S, p.K198E, IVS2 + 1 G > A, *Rec1*) plus p.E326K, we used only studies which performed full sequencing, avoiding the underestimation of other methods of genetic screening. Carrying any pathogenic *GBA1* variant plus p.E326K was associated with an increased risk of PD (OR = 3.51, 95% CI = 1.6–7.4), but heterogeneity was significant (Tarone *p* = 0.02). After excluding the cohort with extreme OR (with two carriers of variants in controls, but none in patients with PD), the heterogeneity was removed (OR = 4.63, 95% CI = 1.9–10.7; Tarone *p* = 0.96; Fig. 1a). For p.L444P (combining full and targeted sequencing studies), the average allelic frequency was 0.028 in patients and 7.7 × 10^−4^ in controls; the variant increased substantially the risk of PD (OR = 20.2, 95% CI = 3.4–118.9, Fig. 1b), and heterogeneity was not significant (Tarone *p* = 0.39). For p.N370S (combining full and targeted sequencing studies), the average allelic frequency was 0.012 in patients and 0.001 in controls; the variant increased the risk of PD (OR = 4.9, 95% CI = 1.2–19.8, Fig. 1c); however, the heterogeneity was significant (Tarone *p* = 0.02). We must highlight that the p.L444P variant causes a more severe form of Gaucher’s disease than p.N370S^6^. Only one study (including individuals from Peru and Colombia) explored the effect of pathogenic variants of *GBA1* on clinical phenotype (age at onset), and carriers had motor symptoms approximately eight years earlier than non-carriers^7^.

**Fig. 1:**
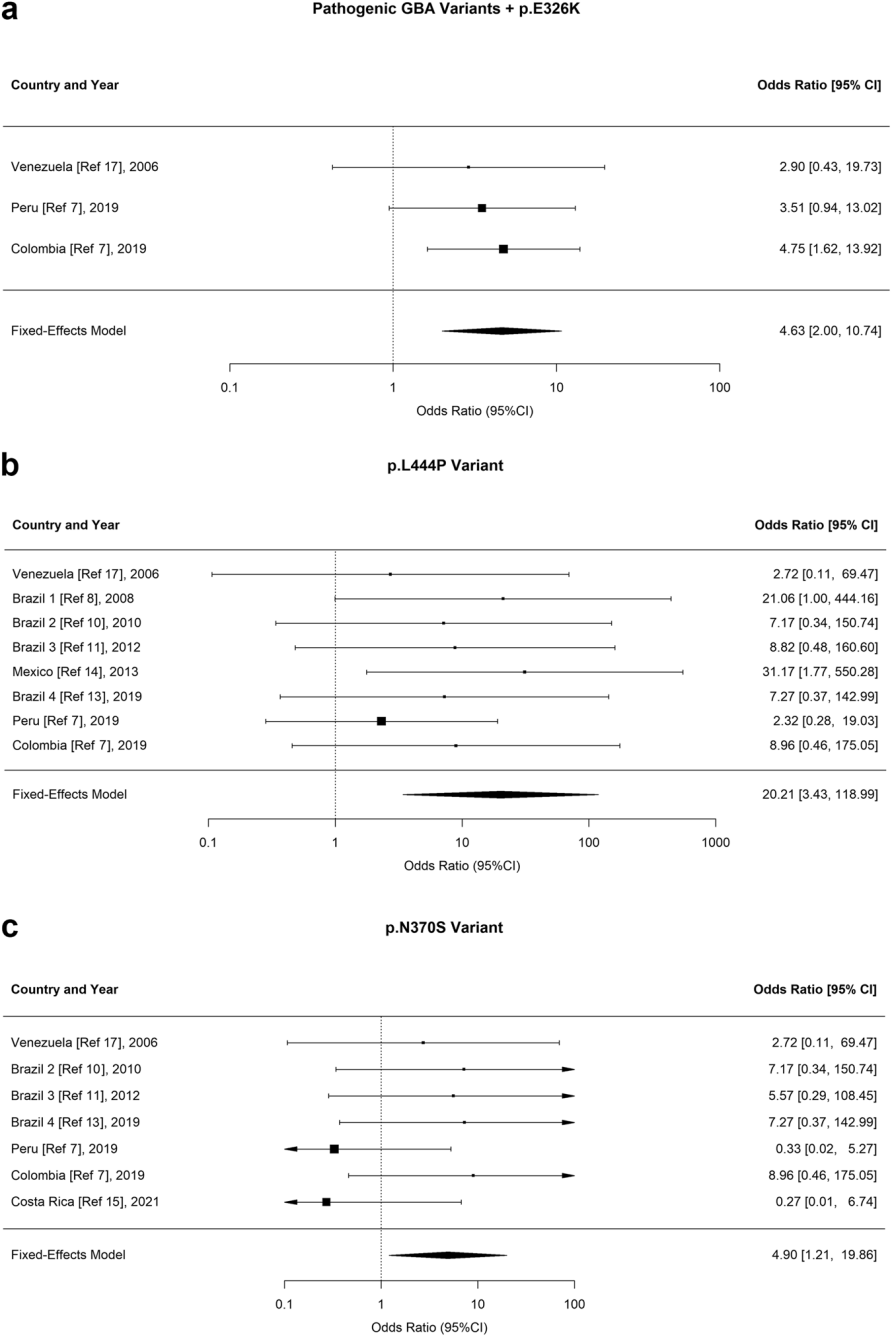
Meta-analyses of *GBA1* variants in patients with Parkinson’s disease from Latin America. **a** Meta-analysis of combined *GBA1* pathogenic variants plus p.E326K from studies which performed full sequencing of the gene. **b** Meta-analysis of the *GBA1* L444P variant. **c** Meta-analysis of the *GBA1* N370S variant.

Until now, approximately 10,000 patients with PD have been full-sequenced for *GBA1* in Europe^5^. The low proportion of variants in controls could be reduced with the full sequencing and larger numbers of participants. The OR values calculated by our meta-analysis for combined pathogenic *GBA1* variants plus p.E326K and p.N370S were similar to previous studies in non-Ashkenazi populations (Supplementary Table 1). However, the overall OR for p.L444P in Latin America was one of the highest values in non-Ashkenazi populations (Supplementary Table 1), mainly due to studies from Brazil^8–13^ and Mexico^14^. We detected heterogeneity between studies, probably due to low numbers and distinct inclusion criteria (studies recruiting any patients with sporadic PD, focusing on patients with early-onset PD or positive family history of PD). For example, we excluded the study from Costa Rica from the meta-analysis to reduce heterogeneity. The study found no *GBA1* variants in patients with PD, probably due to low sample size, the method of sequencing (molecular inversion probes), and a geographical enrollment bias (participants from a specific metropolitan area, in the detriment of metropolitan areas and coastal zones of the country)^15^. Full sequencing of *GBA1* in Latinos has already discovered the pathogenic p.K198E variant in Colombia, with a prevalence of 6% in patients with PD, increasing the disease risk six-fold^7^, showing that some variants of clinical relevance may only be reported after sequencing the whole gene.

Thus, considering the results of our review, it remains unclear how much the full sequencing of *GBA1* would increase the number of PD-associated variants found compared to targeted approaches in Latin America. The number of studies that performed full sequencing of *GBA1* is very low (four cohorts) and included a limited number of patients compared to other populations. More large-scale studies in PD-*GBA* using the full sequencing in Latin America are needed and may elucidate this issue. Promising ongoing clinical trials on PD-*GBA* may bring novel therapies for these patients; providing more *GBA1* full-sequencing opportunities for Latin American populations would diminish health disparities for underrepresented communities.

## Methods

We performed a search of PubMed/MEDLINE and EMBASE from inception until October 2021. We created search strings for each database using “Parkinson’s disease,” “GBA,” and the countries in Latin America (Supplementary Table 2). Two rounds of study selection were performed. In the first round, we included original studies describing patients with PD carrying *GBA1* variants from all countries of Latin America. Reviews, meta-analyses, and studies with animal models were excluded. In the second round, full texts were evaluated, and we selected articles that reported *GBA1* genotyping (pathogenic variants plus p.E326K variant) or *GBA1* sequencing on cohorts of patients with PD. Two reviewers performed selection rounds independently, and disagreements were resolved by consensus. After, data were collected through an online spreadsheet.

## Supplementary information

Supplemental Material

## Data Availability

The datasets generated and/or analyzed during the current study are available from the corresponding author on reasonable request. Other data are available within the article or supplementary materials.
