# Supplementary material for: Lack of full sequencing GBA1 studies for patients with Parkinson’s disease in Latin America
Source: NPJ Parkinsons Dis. 2022 Aug 8;8:101. doi: 10.1038/s41531-022-00358-z (PMC9360049; doi:10.1038/s41531-022-00358-z)
Supplement: Supplementary file 1 — Supplemental Material [file 41531_2022_358_MOESM1_ESM.pdf]

LACK OF FULL SEQUENCING *GBA1* STUDIES FOR PATIENTS WITH PARKINSON'S DISEASE IN LATIN AMERICA

Bruno Lopes Santos-Lobato, Artur F. Schumacher-Schuh, Ignacio F. Mata

Supplementary Figure 1 Flowchart of the inclusion and exclusion of studies for the meta-analysis

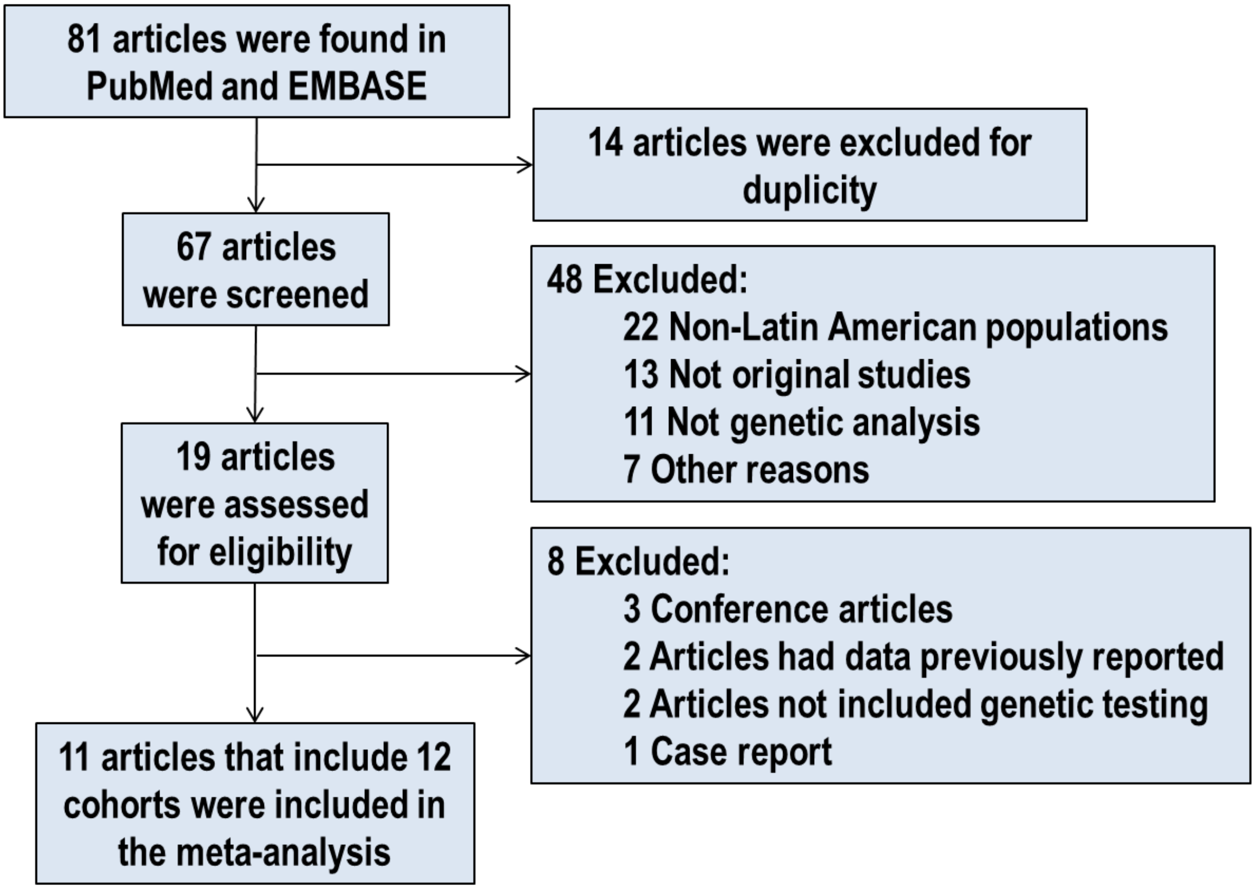

Supplementary Table 1 Odds ratios of Parkinson's disease among carriers of *GBA1* variants in non-Ashkenazi populations reported by previous studies

Author	Year	Country	GBA combined	L444P	N370S
Sidransky et al.[1]	2008	Multicentric	6.51 (3.6-11.7)	8.31 (2.9-23.6)	4.48 (1.2-15.6)
Bras et al.[2]	2009	Portugal	9.2 (2.6-32.4)*	13.25 (0.6-257.5)§	Not calculated
Kalinderi et al.[3]	2009	Greece	6.4 (1.02-39.9)*	3.89 (0.1-81.6)§	3.16 (0.7-13.3)§
Mitsui et al.[4]	2009	Japan	28 (7.3-238.3)	17.58 (1.01-305.3)§	Not calculated
Lesage et al.[5]	2011	France	6.5 (2.5-19.2)	Not calculated	6.22 (1.4-25.9)
Lesage et al.[6]	2011	North Africa	8.56 (1.07-68.2)	4.61 (0.2-96.6)§	Not calculated
Choi et al.[7]	2012	South Korea	20.6 (1.2-356.4)	5.29 (0.2-110.6)§	4.61 (0.2-96.6)§
Han et al.[8]	2015	Canada	4.83 (2.5-9.1)*	4.49 (0.2-84.1)§	6.37 (1.3-30.7)§
Crosiers et al.[9]	2016	Belgium	12.43 (2.2-68.1)	8.17 (0.9-73.4)§	Not calculated
Jesús et al.[10]	2016	Spain	2.96 (Not informed)	2.24 (0.84-5.83)§	11.31 (0.6-205.6)§
Ran et al.[11]	2016	Sweden	1.3 (0.9-1.8)§	9.33 (2.04-42.6)§	0.66 (0.03-16.5)§
Emelyanov et al.[12]	2018	Russia	6.93 (0.9-53.1)	Not informed	Not informed
Lunde et al.[13]	2018	Norway	1.57 (0.7-3.3)*§	3.05 (0.1-75.2)§	3.05 (0.12-75.2)§
Lunde et al.[13]	2018	Scotland	1.22 (0.3-4.08)*§	1.45 (0.09-23.4)§	0.48 (0.02-11.8)§
Den Heijer et al.[14]	2020	Netherlands	3.4 (1.8-6.5)	Not calculated	2.9 (0.7-12.2)
Graham et al.[15]	2020	New Zealand	2.43 (0.5-10.7)§	Not calculated	3.01 (0.3-29.04)§
Biswas et al.[16]	2021	India	Not informed	6.15 (0.2-128.7)§	2.47 (0.1-51.9)§
**Present study**	**2022**	**Multicentric**	**4.63 (1.9-10.7)***	**20.2 (3.4-118.9)**	**4.9 (1.2-19.8)**

Odds ratios are given with confidence interval 95%. "Not informed" means that odds ratio or data for calculating odds ratio was not reported in studies. "Not calculated" means that odds ratio could not be calculated because frequency was 0 in either group.

* Odds ratio of pathogenic variants of *GBA1* plus p.E326K.

§ Odds ratio was calculated with data reported in studies.

Abbreviations: GBA combined, pathogenic variants of *GBA1* (plus p.E326K in some studies).

Supplementary Table 2 Detailed search strings

Database	Search string	Number of results
PubMed	((("parkinson disease"[MeSH Terms] OR ("parkinson"[All Fields] AND "disease"[All Fields]) OR "parkinson disease"[All Fields] OR "parkinson s disease"[All Fields] OR ("parkinson disease"[MeSH Terms] OR ("parkinson"[All Fields] AND "disease"[All Fields]) OR "parkinson disease"[All Fields])) AND ("GBA"[All Fields] OR ("glucocerebrosidases"[All Fields] OR "glucosylceramidase"[MeSH Terms] OR "glucosylceramidase"[All Fields] OR "glucocerebrosidase"[All Fields]))) NOT ("systematic review"[Publication Type] OR "review"[Publication Type] OR "letter"[Publication Type] OR "editorial"[Publication Type])) AND "humans"[MeSH Terms] AND ("mexico"[MeSH Terms] OR "mexico"[All Fields] OR "mexico s"[All Fields] OR "mexicos"[All Fields] OR ("guatemala"[MeSH Terms] OR "guatemala"[All Fields] OR "guatemala s"[All Fields]) OR ("honduras"[MeSH Terms] OR "honduras"[All Fields]) OR ("belize"[MeSH Terms] OR "belize"[All Fields]) OR ("el salvador"[MeSH Terms] OR ("el"[All Fields] AND "salvador"[All Fields]) OR "el salvador"[All Fields]) OR ("nicaragua"[MeSH Terms] OR "nicaragua"[All Fields] OR "nicaragua s"[All Fields]) OR ("costa rica"[MeSH Terms] OR ("costa"[All Fields] AND "rica"[All Fields]) OR "costa rica"[All Fields]) OR ("panama"[MeSH Terms] OR "panama"[All Fields] OR "panama s"[All Fields]) OR ("cuba"[MeSH Terms] OR "cuba"[All Fields]) OR ("colombia"[MeSH Terms] OR "colombia"[All Fields] OR "colombia s"[All Fields]) OR ("venezuela"[MeSH Terms] OR "venezuela"[All Fields] OR "venezuela s"[All Fields]) OR ("ecuador"[MeSH Terms] OR "ecuador"[All Fields] OR "ecuador s"[All Fields]) OR ("peru"[MeSH Terms] OR "peru"[All Fields]) OR ("bolivia"[MeSH Terms] OR "bolivia"[All Fields]) OR ("brazil"[MeSH Terms] OR "brazil"[All Fields] OR "brazil s"[All Fields] OR "brazils"[All Fields]) OR ("paraguai"[All Fields] OR "paraguay"[MeSH Terms] OR "paraguay"[All Fields]) OR ("chile"[MeSH Terms] OR "chile"[All Fields] OR "chiles"[All Fields] OR "chile s"[All Fields]) OR ("argentina"[MeSH Terms] OR "argentina"[All Fields] OR "argentina s"[All Fields] OR "argentinae"[All Fields]) OR ("uruguai"[All Fields] OR "uruguay"[MeSH Terms] OR "uruguay"[All Fields] OR "uruguay s"[All Fields]))	15 results
EMBASE	'parkinson disease' AND ('gba gene' OR glucosylceramidase) AND (mexico OR guatemala OR honduras OR belize OR (el AND salvador) OR nicaragua OR (costa AND rica) OR panama OR cuba OR colombia OR venezuela OR ecuador OR peru OR bolivia OR brazil OR paraguay OR chile OR argentina OR uruguay)	66 results

Supplementary References

1. Sidransky, E. et al. Multicenter analysis of glucocerebrosidase mutations in Parkinson's disease. N. Engl. J. Med. 361, 1651–1661 (2009).

2. Bras, J. et al. Complete screening for glucocerebrosidase mutations in Parkinson disease patients from Portugal. Neurobiol. Aging 30, 1515–1517 (2009).

3. Kalinderi, K. et al. Complete screening for glucocerebrosidase mutations in Parkinson disease patients from Greece. Neurosci. Lett. 452, 87–89 (2009).

4. Mitsui, J. et al. Mutations for Gaucher disease confer high susceptibility to Parkinson disease. Arch. Neurol. 66, 571–576 (2009).

5. Lesage, S. et al. Large-scale screening of the Gaucher's disease-related glucocerebrosidase gene in Europeans with Parkinson's disease. Hum. Mol. Genet. 20, 202–210 (2011).

6. Lesage, S. et al. Mutations in the glucocerebrosidase gene confer a risk for Parkinson disease in North Africa. Neurology vol. 76 301–303 (2011).

7. Choi, J. M. et al. Association of mutations in the glucocerebrosidase gene with Parkinson disease in a Korean population. Neurosci. Lett. 514, 12–15 (2012).

8. Han, F. et al. Mutations in the glucocerebrosidase gene are common in patients with Parkinson's disease from Eastern Canada. Int. J. Neurosci. 126, 415–421 (2016).

9. Crosiers, D. et al. Mutations in glucocerebrosidase are a major genetic risk factor for Parkinson's disease and increase susceptibility to dementia in a Flanders-Belgian cohort. Neurosci. Lett. 629, 160–164 (2016).

10. Jesús, S. et al. GBA Variants Influence Motor and Non-Motor Features of Parkinson's Disease. PLoS One 11, e0167749 (2016).

11. Ran, C. et al. Strong association between glucocerebrosidase mutations and Parkinson's disease in Sweden. Neurobiol. Aging 45, 212.e5–212.e11 (2016).

12. Emelyanov, A. K. et al. Mutation analysis of Parkinson's disease genes in a Russian data set. Neurobiol. Aging 71, 267.e7–267.e10 (2018).

13. Lunde, K. A. et al. Association of glucocerebrosidase polymorphisms and mutations with dementia in incident Parkinson's disease. Alzheimers. Dement. 14, 1293–1301 (2018).

14. den Heijer, J. M. et al. A Large-Scale Full GBA1 Gene Screening in Parkinson's Disease in the Netherlands. Mov. Disord. 35, 1667–1674 (2020).

15. Graham, O. E. E. et al. Nanopore sequencing of the glucocerebrosidase (GBA) gene in a New Zealand Parkinson's disease cohort. Parkinsonism Relat. Disord. 70, 36–41 (2020).

16. Biswas, A. et al. Identification of GBA mutations among neurodegenerative disease patients from eastern India. Neurosci. Lett. 751, 135816 (2021).
